# Systemic Reprogramming of Endothelial Cell Signaling in Metastasis and Cachexia

**DOI:** 10.1152/physiol.00001.2023

**Published:** 2022-07-01

**Authors:** Stephanie F. Preuss, Denise Grieshober, Hellmut G. Augustin

**Affiliations:** ^1^European Center for Angioscience (ECAS), Medical Faculty Mannheim, Heidelberg University, Mannheim, Germany; ^2^Division of Vascular Oncology and Metastasis, German Cancer Research Center (DKFZ-ZMBH Alliance), Heidelberg, Germany; ^3^Faculty of Biosciences, Heidelberg University, Heidelberg, Germany

**Keywords:** angiocrine signaling, cachexia, metastasis, systemic signaling, vascular endothelium

## Abstract

Proliferating cancer cells secrete a multitude of factors impacting metabolism, interorgan communication, and tumor progression. The distribution of tumor-derived factors to distant organs occurs via the circulation, which provides an extensive reactive surface lined by endothelial cells. Primary tumor-derived proteins impact cancer progression by modulating endothelial cell activation at the (pre-)metastatic niche, which affects tumor cell dissemination as well as the outgrowth of seeded metastatic cells into overt tumors. In addition, new insight indicates that endothelial cell signaling contributes to metabolic symptoms of cancer, including cancer-associated cachexia, opening a new field of vascular metabolism research. This review addresses how tumor-derived factors systemically affect endothelial cell signaling and activation and impact distant organs as well as tumor progression.

## Introduction

The notion that endothelial cells (ECs) are merely inactive, at best responsive cells lining the inner surface of blood vessels, has fundamentally changed in recent years. Instead, ECs are increasingly recognized as highly dynamic instructive regulators and modulators of the local microenvironment, creating a unique vascular niche during homeostatic and pathological conditions (1, 2). Recent breakthrough discoveries have further advanced the concepts of angiocrine signaling, EC-derived instructive signals, beyond locally acting paracrine signaling mechanisms toward systemic signaling pathways communicating through the circulation as a regulator of systemic homeostasis (3, 4).

**FIGURE 1.**
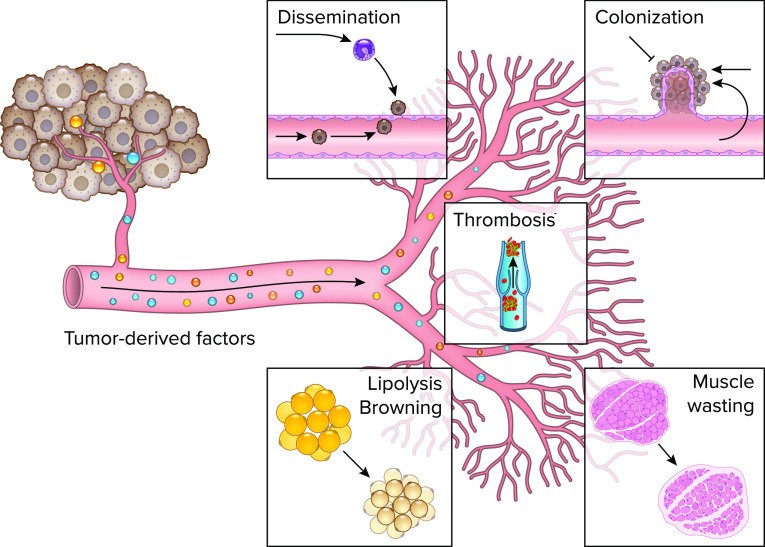
**Primary tumor-derived factors systemically affect the distant vasculature to regulate metastasis, thrombosis, and cachexia** Primary tumor-derived factors change endothelial cell signaling at the pre-metastatic niche to promote tumor cell dissemination and modulate angiogenesis in metastatic nodules, which affects tumor colonization. Additionally, tumor-derived factors may cause changes in organ function of cancer patients and induce thrombosis in the distant vasculature. During cachexia, cancer cells may regulate endothelial cell signaling to induce adipocyte wasting by activating lipolysis and browning. In skeletal muscle, tumor-derived factors may cause proteolysis and impaired regeneration via abnormal angiocrine signaling. Image was created with BioRender.com, with permission.

The importance of ECs within the tumor stroma has been highlighted in numerous authoritative reviews in recent years (5, 6). ECs do not only nourish the primary tumor by growing new blood vessels upon angiogenic stimulation but provide a platform for secreted and membrane-bound factors, which interact with cancer cells and contribute to the formation of a growth-promoting tumor-microenvironment. Beyond its local regulatory role, EC-derived angiocrine signaling at distant sites can also affect organ function. Notably, primary tumor-secreted factors, which can act far beyond the tumor boundaries, may systemically affect ECs of distant organs (7). The importance of studying the systemic effects of tumor cells is highlighted by the fact that most cancer-related deaths are due to the systemic secondary impact of the primary tumor such as metastasis, cachexia, and thrombosis (8–10). The effects of modified angiocrine signaling during tumor progression have been primarily studied in the context of the metastatic site; however, the vasculature also impacts processes like coagulation, inflammation, and metabolism, leading to thrombosis and cachexia (FIGURE 1). Thus, studying cancer as a systemic disease has in recent years become an important focus of research to identify and validate novel targets for cancer therapy.

In this review, the systemic effects of tumor-derived factors on distant endothelial beds will be described. We propose the concept that ECs should be considered as independent drivers of disease progression impacting the homeostatic signaling of distant organs in response to a systemic trigger, e.g., cancer-derived factors. Angiocrine signaling modulates the host’s macroenvironment by inducing numerous organ-specific microenvironmental changes.

## The Effect of Primary Tumor-Secreted Factors on ECs at the Pre-metastatic Site

Metastatic dissemination can be portrayed as a multistep succession of events that eventually leads to the outgrowth of metastatic nodules in distant tissues. As part of this process, cancer cells intravasate into nearby vessels and enter the circulation (11, 12). Subsequently, these circulating tumor cells (CTCs) enter secondary organ sites as disseminated tumor cells that may form metastases. However, these intricate steps critically depend on the local microenvironment, which determines whether tumor cell dissemination can take place. To prepare pre-metastatic sites for tumor cells, primary tumors secrete certain factors, mobilize bone marrow-derived cells (BMCs), and act on the immune system. This enables the formation of a supportive and receptive tissue microenvironment, the so-called pre-metastatic niche (13).

Cellular and molecular dissection of the pre-metastatic niche has revealed that tumor-derived factors can promote metastatic spread in specific, distant organs. Notably, primary tumor-derived vascular endothelial growth factor (VEGF) and placental growth factor (PlGF) contribute to the recruitment of CD11b^+^ myeloid cells to the lungs before tumor dissemination in Lewis lung carcinoma (LLC) and B16F10 melanoma preclinical mouse models. This results in enhanced recruitment of metastatic cells to the lungs by providing a permissive niche for incoming tumor cells (14). Building on these early landmark findings, the significance of the pre-metastatic niche was studied in more detail in subsequent years and key characteristics of the niche, including immunosuppression, inflammation, angiogenesis, and vascular permeability, have been described (13).

Tumor cells secreting the proinflammatory cytokine IL-1β trigger a granulocyte-colony stimulating factor (G-CSF)-dependent expansion and polarization of neutrophils. Neutrophils thereby acquire the ability to suppress cytotoxic CD8^+^ T cells, which in turn promotes metastasis formation (15). Moreover, an aggregation of neutrophils was detected in peripheral organs of mammary carcinoma and insulinoma-bearing mice with an accumulation of proinflammatory cytokines such as IL-1β, IL-6, and CXCL1, which contribute to the formation of the pre-metastatic niche (16). The effect of tumor-derived factors on inflammation and immunosuppression has been reviewed in detail elsewhere (13). This review will focus on the effect on ECs and the effector functions of ECs in the target organ.

To promote metastasis, primary tumors may stimulate vascular permeability and angiogenesis in the pre-metastatic niche before the arrival of metastatic tumor cells. This can be mediated via soluble factors or exosomes that are secreted by the primary tumor (13). VEGF, PlGF, transforming growth factor β (TGF-β), tumor necrosis factor α (TNF-α), and G-CSF are some of the most important factors, which have been described to be secreted by the primary tumor and to affect ECs in secondary organs. Primary tumor-derived VEGF specifically upregulates matrix metalloproteinase 9 (MMP9) in pre-metastatic lung ECs via VEGFR-1/Flt-1 tyrosine kinase and thereby promotes lung metastasis by enhancing tumor cell invasion (17). In addition, primary tumor-secreted VEGF may induce EC hyperpermeability in the lungs by accelerating occludin degradation leading to the disruption of tight junctions (18). Subsequently, the primary tumor may induce an increase in total vessel density in the pre-metastatic lung. This effect is mediated by systemically circulating VEGF, which leads to an elevated prostaglandin E2 production in mouse pulmonary microvascular ECs and thereby enhances the adhesion of metastatic 4T1 breast cancer cells (19). In addition to VEGF, PlGF is secreted by the primary tumor and can induce foci of vascular hyperpermeability in the lungs by upregulating E-selectin via FAK in ECs (20). Other factors that were shown to be induced by the primary tumor-expressed proteins VEGF, TGF-β, and TNF-α are the inflammatory chemoattractants S100A8 and S100A9. In the premetastatic lung, they can trigger the accumulation of macrophage antigen 1 (Mac1)-positive myeloid cells (21, 22). Mechanistically, S100A8 and S100A9 induce serum amyloid A 3 (SAA3), which stimulates NF-κB signaling in ECs in a Toll-like receptor 4-dependent manner and thereby facilitates metastasis (23). Further, G-CSF was shown to be overexpressed in several metastatic tumors and to expand Ly6G- and Ly6C-positive granulocytes in the premetastatic niche. These cells produce the Bv8 protein, which is implicated in angiogenesis and stimulates tumor cell migration through activation of the prokineticin receptor 1 (PKR-1) (24).

Despite secreting soluble factors to form a premetastatic niche, primary tumors can also disseminate their cargo in exosomes (25). This was first described in melanoma cells, which release exosomes to prepare sentinel lymph nodes for metastasis (26). Later, it was shown in a pancreatic ductal adenocarcinoma (PDAC) mouse model that tumor-derived exosomes can enhance metastatic burden in the liver. This effect is mediated by macrophage migration inhibitory factor (MIF), which is expressed in exosomes and increases macrophage recruitment from the bone marrow (27). In addition, EC-specific effects of tumor-derived exosomes were reported. In renal cell carcinoma, a subset of tumor-initiating cells (CD105^+^) releases exosomes, which trigger angiogenesis and promote the formation of a premetastatic niche. These exosomes contain proangiogenic mRNAs and miRNAs that specifically enhance VEGF, MMP2, and VEGFR1 expression in lung ECs (28). These molecules were previously shown to be essential for the formation of a lung premetastatic niche (17, 29). In addition, proteomics analysis of ovarian cancer exosomes revealed activating transcription factor 2 (ATF2) and metastasis-associated protein 1 (MTA1) as exosome-derived proteins that upregulate angiogenesis (30). Furthermore, soluble E-cadherin is released from ovarian cancer in exosomes and induces angiogenesis. Mechanistically, soluble E-cadherin heterodimerizes with VE-cadherin on ECs and thereby activates β-catenin and NF-κB signaling (31). A three-dimensional in vitro system to study the effect of primary tumor-derived factors on the premetastatic niche was introduced in the form of a liver-on-a-chip. By exploiting this system, breast cancer-derived extracellular vesicles were shown to induce endothelial-to-mesenchymal transition (EMT) in liver sinusoidal endothelial cells and destabilize the endothelial barrier, which facilitates metastatic dissemination in the liver (32).

**FIGURE 2.**
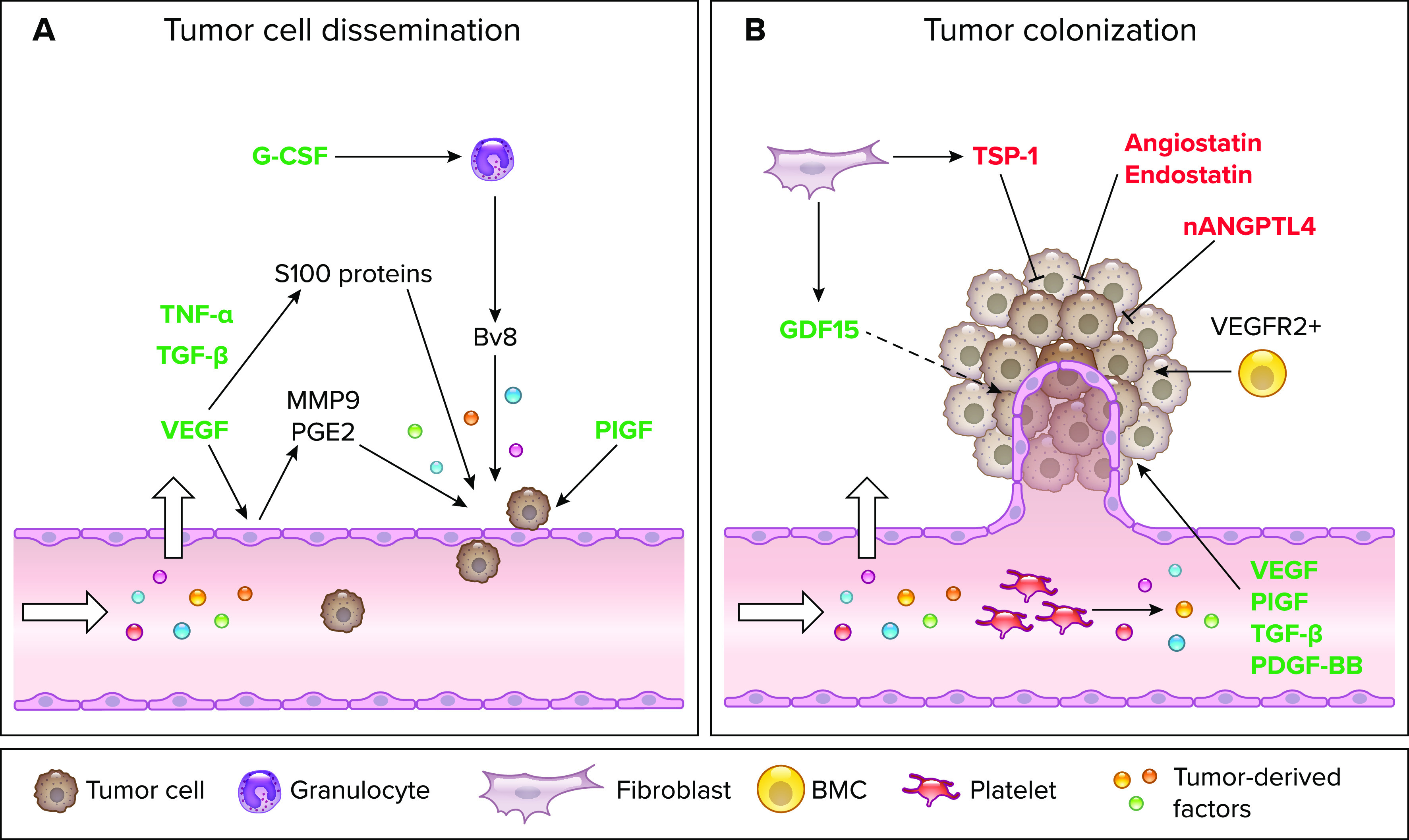
**Primary tumor-derived factors affect tumor cell dissemination and colonization at distant sites** *A*: graphical illustration of the effects of primary tumor-derived factors on endothelial cells (ECs) at the premetastatic niche. VEGF, TNF-α, transforming growth factor β (TGF-β), and placental growth factor (PlGF) act directly on ECs at the pre-metastatic niche and thereby induce EC hyperpermeability and enhance tumor cell dissemination. Granulocyte-colony stimulating factor (G-CSF) affects transmigration indirectly via expanding Ly6G^+^Ly6C^+^ granulocytes, which activate ECs by secreting Bv8. *B*: Schematic representation of the effects of primary tumor-derived factors on ECs at the metastatic site. VEGF, PlGF, TGF-β, and PDGF-BB are secreted by tumor-educated platelets and induce angiogenesis thereby promoting tumor colonization. VEGFR2^+^ bone marrow-derived cells (BMCs) are enhanced and contribute to these processes. Cancer-associated fibroblasts (CAFs) from the primary tumor secrete growth differentiation factor 15 (GDF15) and thrombospondin-1 (TSP-1), which enhance or reduce angiogenesis at the metastatic site, respectively. Molecules such as angiostatin, nANGPTL4, and endostatin are secreted by the primary tumor and directly act on ECs to reduce sprouting and thereby inhibit the outgrowth of metastatic nodules. Tumor-derived factors promoting cancer cell dissemination or colonization are depicted in green and tumor-derived factors inhibiting these processes are highlighted in red. Image was created with BioRender.com, with permission.

Taken together, several soluble factors or exosomes can, either directly or via the stimulation of immune cells, enhance angiogenesis and vascular permeability at the premetastatic site and thereby prepare distant organs for metastatic colonization (FIGURE 2A).

## The Effect of Primary Tumor-Secreted Factors on Tumor ECs at the Metastatic Site

The final step of the metastatic cascade involves the outgrowth and propagation of disseminated tumor cells into overt tumors at the metastatic site. To form macroscopic metastases, the microenvi-ronment needs to be adapted to favor remodeling of the extracellular matrix, immune cell inhibition, and angiogenesis (7). There have been a number of publications reporting primary tumor-derived angiogenic factors which promote metastatic outgrowth (7, 33, 34). For example, in a luminal breast cancer model, primary tumors can modulate the systemic macroenvironment by loading circulating platelets with a repertoire of proangiogenic cytokines such as VEGF, TGF-β1, PDGF-BB, and PIGF. Subsequently, proangiogenic platelets accumulate at sites of indolent tumors where they aid vessel formation. In addition, in the presence of a primary tumor, the expression of proangiogenic VEGFR2 is enhanced on BMCs from the bone marrow, which promotes the growth of disseminated tumor cells into highly vascularized tumors (33).

A recent report has highlighted for the first time that cancer-associated fibroblasts (CAFs) promote the dissemination of tumor cells. Within this publication, the prostate cancer tumor microenvironment was shown to have a significantly higher expression of growth differentiation factor 15 (GDF15) as compared to the healthy prostate microenvironment. This was attributed to the higher expression of GDF15 in CAFs. Moreover, GDF15-expressing fibroblasts were shown to exert systemic effects on the outgrowth of distant, otherwise indolent, prostate cancer cells (34). Proangiogenic functions of GDF15 have previously been reported in different cell types. For example, in hepatocellular carcinoma, GDF15 acts via AKT, MAPK, and NF-κB signaling to enhance EC proliferation and capillary-like tube formation ability (35). Whether CAF-derived GDF15 promotes the outgrowth of distant micrometastases via a similar mechanism remains to be investigated.

In contrast, there are also a number of publications showing that the primary tumor secretes factors that inhibit the outgrowth of secondary tumor implants and metastasis (36). This phenomenon is referred to as concomitant tumor resistance (CTR) and was first described in 1906 by Ehrlich and Apolant (37). CTR has been experimentally studied in mouse models of melanoma (38, 39), breast cancer (40, 41), lung cancer (42, 43), lymphoma, and fibrosarcoma (44, 45). Different hypotheses have been proposed to explain the phenomenon of CTR. According to these hypotheses, the antimetastatic effects may be due to either the host’s immune system (immunological hypothesis) or systemically acting primary tumor-released antimitogenic and/or antiangiogenic cytokines (angiogenesis-based hypothesis). According to the immunological hypothesis, the primary tumor generates an antitumor immune response that inhibits the outgrowth of a secondary tumor implant (36). This hypothesis has been experimentally demonstrated in mouse models of melanoma and breast cancer. Hence, in a B16F10 melanoma mouse model, the primary tumor was shown to reduce metastasis formation of intravenously injected tumor cells by changing the immune landscape (38) and by reducing circulating platelets (39). In addition, in an EMT6 mammary carcinoma model, the primary tumor was shown to inhibit the metastatic outgrowth of CTCs via activation of CD8+ T cells (40). However, the immunological hypothesis does not provide a compelling explanation for the fact that CTR is also observed in nonimmunogenic tumors (46).

A nonimmunological explanation of CTR relies on the release of antiangiogenic factors by the primary tumor. The angiogenesis-based hypothesis was first proposed by O’Reilly et al. (42). In 1994, they identified the antiangiogenic, primary tumor-secreted factor angio-statin in an LLC mouse model and discovered that the lack of angiostatin after surgical removal of the primary tumor is responsible for the rapid outgrowth of distant metastasis. Mechanistically, angio-statin is supposed to inhibit EC proliferation at the metastatic site, which reduces angiogenesis (42). Subsequent studies identified another antiangiogenic factor, named endostatin, as a primary tumor-secreted factor that suppresses the growth of metastases when administered systemically (47). Endostatin is a proteolytic fragment of type XVIII collagen that induces EC apoptosis by inhibition of cyclin D1. It binds to integrin α5β1 on the surface of ECs, which downregulates the activity of RhoA GTPase leading to the disassembly of the actin cytoskeleton and thereby suppression of angiogenesis (48). Yet another angiogenesis-regulating molecule, thrombospondin-1 (TSP-1), is secreted by a fibrosarcoma primary tumor model in mice and has been proposed to inhibit angiogenesis in the cornea and the growth of metastasis (44). Following these early discoveries, Folkman proposed the contextuality of angiogenic molecules, whereby proangiogenic molecules were believed to be short-lived and locally acting, whereas antiangiogenic molecules were proposed to be long-lived and systemically acting (49, 50). This hypothesis may indeed provide an explanation for the central paradox of CTR: how can a primary tumor-secreted cytokine inhibit angiogenesis at the metastatic site, while having no effect on the primary tumor vasculature itself (36, 51)? According to Hanahan and Folkman (49), the primary tumor-secreted proangiogenic factors stay local, counteract the antiangiogenic molecules and thereby allow an angiogenic switch in the local tumor microenvironment.

Following an intense phase of hype, the field of CTR regulating molecules has made little progress in the last 20 years. This is primarily due to the fact that much of this early work did not stand the test of time and because supposedly CTR-regulating molecules proved translationally disappointing, even though genetic experiments, for example in TSP-1- or collagen XVIII-deficient mice, revealed compelling evidence for angiogenesis-regulating functions of these molecules(52–54).

The concepts of CTR have seen a recent revival with the landmark discovery showing that the primary tumor-derived NH_2_-terminal fragment of angiopoietin-like 4 (nANGPTL4) inhibits metastasis by reducing vascularity at the metastatic site (55). Mechanistically, nANGPTL4 downregulates Wnt signaling via the syndecan 4 (SDC4) receptor. Conversely, the COOH-terminal fragment cANGLPTL4 stimulates angiogenesis in the primary tumor in an integrin-dependent manner. As ANGPTL4 binds to integrins via the COOH-terminal fragment, the extracellular matrix serves as a reservoir and locally restricts cANGPTL4 to the primary tumor, whereas nANGPTL4 is present systemically. Differential pro- and antiangiogenic effects of the C- and the NH_2_-terminal fragments, regulated by differential receptor engagement, are in the case of ANGPTL4 regulated by differential expression and presentation of receptors and ligands. Notably, while the proangiogenic COOH-terminal fragment can primarily be detected in primary tumors, the NH_2_-terminal fragment is predominately detected in the circulation in both tumor-bearing mice and men (55).

Clearly, much more needs to be learned about the mechanisms of CTR, and it is worth reconsidering some of the older literature. For example, by comparing several mouse models of CTR, Franco et al. (45) observed that CTR appears in two peaks. The first peak occurs when the primary tumor is small and is proportional to tumor immunogenicity. The second peak appears in large tumors and does not correlate with tumor immunogenicity. However, it correlates with the activity of serum factors that inhibit the proliferation of tumor cells. This effect is not solely restricted to metastasis. It was shown that a larger primary tumor inhibits the growth of a smaller primary tumor depending on the ratio between tumor sizes: A high ratio leads to inhibition of the smaller tumor and a small ratio leads to stimulation of tumor growth (56).

Importantly, the preclinical findings of CTR are supported by the clinical observation that metastasis occurs often only after the primary tumor has been resected. For instance, pulmonary metastases were shown to be increased in patients with osteosarcoma after primary tumor removal due to an increase in angiogenesis (57). In addition, a rapid metastatic progression is observed in patients with bladder cancer upon radical cystectomy. This was shown to be due to the sudden lack of antiproliferative molecules such as endostatin, angiostatin, and TSP-1, which are highly expressed in human bladder cancer (58). In colorectal cancer patients, metastatic colonization and vascular density in the liver were shown to be increased upon primary tumor removal (59). Overall, these clinical observations validate the preclinical findings that the primary tumor actively limits the growth of metastasis by secreting antiangiogenic cytokines.

As the primary tumor has an effect on metastatic dissemination and colonization, systemic mechanisms must be more critically considered in cancer therapy. Clinical observations suggest that current treatment strategies may enhance systemic changes and eventually promote metastatic colonization. For example, G-CSF is used in cancer patients with myelosuppression induced by chemotherapy. However, as already mentioned, G-CSF induces both a neutrophil-dependent suppression of CD8^+^ T cells and an expansion of Ly6G^+^Ly6C^+^ granulocytes, which enhances tumor cell extravasation and angiogenesis in the pre-metastatic niche, respectively (60). In addition, antiangiogenic therapies can induce the systemic upregulation of off-target molecules such as osteopontin and G-CSF and thereby increase the extravasation potential of tumor cells or support the pre-metastatic niche. Specifically, VEGF receptor tyrosine kinase inhibitors were shown to enhance BMC recruitment to distant organs, inflammatory pathway activation, and altered EC adhesion molecule function, which eventually leads to changes in the endothelial microenvironment and increases extravasation potential for tumor cells (61). Overall, both pro- and antiangiogenic factors have been reported to be secreted by primary tumors to favor or limit metastatic outgrowth, respectively (FIGURE 2B). To optimize strategies to inhibit metastatic progression, it is essential to gain a more detailed molecular and mechanistic understanding of the interplay of the primary tumor with ECs at the meta-static site in different contexts. Toward this end, it is important to compare circulating factors in the serum of healthy versus tumor-bearing patients and to further study their effect on endothelial cells at the metastatic site.

## Tumor-Induced Effects on Organs That Do Not Represent the Metastatic Site

While the majority of the scientific literature focuses on the effects of primary tumor-derived factors on metastatic sites, especially the lungs, there is much less known about the effects on the vasculature in organs that do not represent metastatic sites. However, there is solid clinical evidence that patients with cancer have a significantly increased risk of developing thrombosis. This effect is seen in many cancer types such as colorectal, pancreatic, and gastric cancer, a phenomenon commonly referred to as Trousseau syndrome (9). This has been attributed to cancer cells releasing procoagulant and fibrinolytic proteins as well as inflammatory cytokines. In addition, cancer cells are capable of directly adhering to host cells, including ECs, thereby stimulating prothrombotic properties. Eventually, some of these mechanisms, such as fibrin formation and deposition, not only promote thrombotic events but also stimulate tumor progression by creating a more favorable niche (62).

Tissue factor (TF) is the primary cellular initiator of the blood coagulation cascade, which leads to the generation of thrombin with the subsequent formation of a fibrin clot. In many tumor types, TF expression was shown to be induced. In colorectal cancer (CRC), mutations in K-ras and p53 result in constitutive activation of MAPK and phosphatidylinositol 3-kinase (PI3K) signaling, which leads to enhanced TF expression. TF affects ECs via different mechanisms. Results from mouse models of CRC revealed that TF can directly impact tumor angiogenesis through the modulation of VEGF and thrombospondin levels in ECs (63). In addition, alternatively spliced TF activates α6β1 and αVβ3 integrins on ECs and acts as a proangiogenic stimulus by activating focal adhesion kinase PI3K, MAPK, and Akt (64). Thereby, TF triggers angiogenesis in a paracrine fashion by targeting vascular cells. It also induces cytoskeleton remodeling in ECs by stimulating Rac1 and p38 expression leading to enhanced migration of ECs (65). Despite releasing soluble TF, tumor cells can also release exosomes and microparticles containing TF. These microparticles bind to sites of vascular injury and enhance thrombosis (66).

In addition to thrombosis, some cancer patients show limited organ function such as impaired heart and kidney function, which is due to vascular dysfunction. This phenomenon was attributed to the formation of neutrophil extracellular traps (NETs). NETs are extracellular networks consisting of DNA released from neutrophils together with antimicrobial peptides and proteases derived from neutrophil granules and play a role in microbial defense (16). In addition, NETs have been implicated in cancer-associated thrombosis, where tumor cell-secreted G-CSF has been shown to promote NETosis in neutrophils (67). NET accumulation leads to an upregulation of proinflammatory adhesion molecules (ICAM-1, VCAM-1, and E-selectin) as well as proinflammatory cytokines (IL-1β and IL-6) in ECs, which can trigger organ failure due to hypo-perfusion of organs in cancer patients (16). In other pathological conditions, NETs can induce endothelial damage and have cytotoxic effects on ECs. Thereby, NET formation provides a possible explanation for tumor-induced organ failure, which is a substantial cause of morbidity in cancer patients.

## Angiocrine Signaling During Cancer-Associated Cachexia

Cancer progression and chronic inflammation lead to massive changes in the body’s metabolism causing an imbalance in energy expenditure. The consequential involuntary body weight loss caused by muscle as well as adipose tissue wasting is defined as cachexia and cannot be completely reversed by dietary supplementation (68). The occurrence of cancer-associated cachexia (CAC) highly depends on the tumor type and correlates with the disease stage (69). Generally, CAC reduces the quality of life and increases morbidity and mortality as well as cancer treatment toxicities (70, 71).

As a multifactorial syndrome, CAC causes changes in many organs and their interorgan and intertissue communication. Muscle wasting is mediated by muscle proteolysis, the degradation of muscle fibers as well as the lack of muscle regeneration due to exhausted muscle stem cells, so-called satellite cells (SCs). The increased expression of the E3 ligases MuFR1 and MAFbx of the ubiquitin-proteasome system is one of the hallmarks of cachectic muscles (72). The metabolic changes in adipose tissue lead to increased lipolysis and adipose tissue browning, which is characterized by an increased expression of the uncoupling protein 1 (UCP1). In adipocytes, UCP1 causes energy wasting due to excessive heat production (73). Thermogenesis is activated in the remaining adult brown adipocytes increasing the energy expenditure. Additionally, the combination of loss of appetite caused by alterations in hypothalamic signaling mechanisms and malabsorption due to changes in the intestinal barrier and the microbiome composition worsens the negative energy balance (74).

Cachexia research has focused on a multitude of organs and tissues at different cachectic stages to comprehend the immense variety of changes caused by cachexia syndrome. Additionally, extensive mouse strain-specific and tumor-specific differences have increased the complexity of CAC research (75). Since ECs are involved in many pathways within tumor progression and the metastatic cascade (as mentioned above), the question arises if ECs influence or are influenced by metabolic conditions like cachexia. Only a few very recent studies have focused on the effect of ECs in cachexia, but in the light of the novelty of this subject, we highlight well-known cachexia markers, which may have an endothelial origin, may change vasculature function, or may lead to altered angiocrine signaling opening a new research direction in the expanding field of CAC.

## The Effect of Cytokines on Endothelial Cells During Cancer-Associated Cachexia

Primary tumors secrete various cytokines that have many different effects on the immune system, the surrounding tissue as well as distant sites. Specifically, increased IL-6, IL-8, and TNF-α concentrations are characteristic of CAC (76). Furthermore, tumor-derived exosomes and vesicles have been described to induce skeletal muscle and adipose tissue wasting (77, 78). Here, we will focus on IL-6 due to its pro-inflammatory autocrine and paracrine function in ECs and its distinct roles in CAC.

During the progression of CAC, chronic inflammation mediated by IL-6 and TNF-α has been described to induce white adipose tissue browning (79) and increased lipolysis mediated by STAT3 signaling and the adipose triglyceride lipase (80). However, a systemic study of 136 patients with PDAC concluded that IL-6 only correlates with cancer stage and not with cachexia (81) conceding that IL-6 may mediate cachexia but should not be considered as a biomarker. Cachectic mice have been described to be hypercoagulable partially due to tumor-derived IL-6, which is inducing thrombin expression (82). Likewise, in skeletal muscle, IL-6 plays a pivotal role during CAC regulating mitochondrial dynamics, biogenesis, function, and mitophagy by inducing STAT3 phosphorylation (83). The direct and indirect effects of IL-6-induced STAT3 signaling in CAC have been previously summarized (84).

Since ECs do not express the membrane-bound IL-6 receptor (IL-6R), the soluble IL-6Rα needs to bind to IL-6 to signal through endothelial gp130 activating the expression of STAT3 target genes, notably the production of chemokines [e.g., monocyte chemoattractant protein-1 (MCP-1)] or adhesion molecules (e.g., E-selectin, ICAM-1, and VCAM-1) (85). This alternative signaling via the soluble receptor is described as trans-signaling. Additionally, upon inflammatory stimuli, ECs produce and secrete large amounts of IL-6 increasing its local and systemic concentrations. Tumor-derived IL-6 induces endothelial IL6-STAT3 signaling, which in turn may lead to increased plasma concentrations of cachexia-promoting factors like C-C motif chemokine ligand 2 (CCL2/MCP-1), commonly described in CAC patients (86, 87). The production of IL-6, IL-10, and PAI-1 in ECs is furthermore triggered by IL-6 signaling itself (88).

**FIGURE 3.**
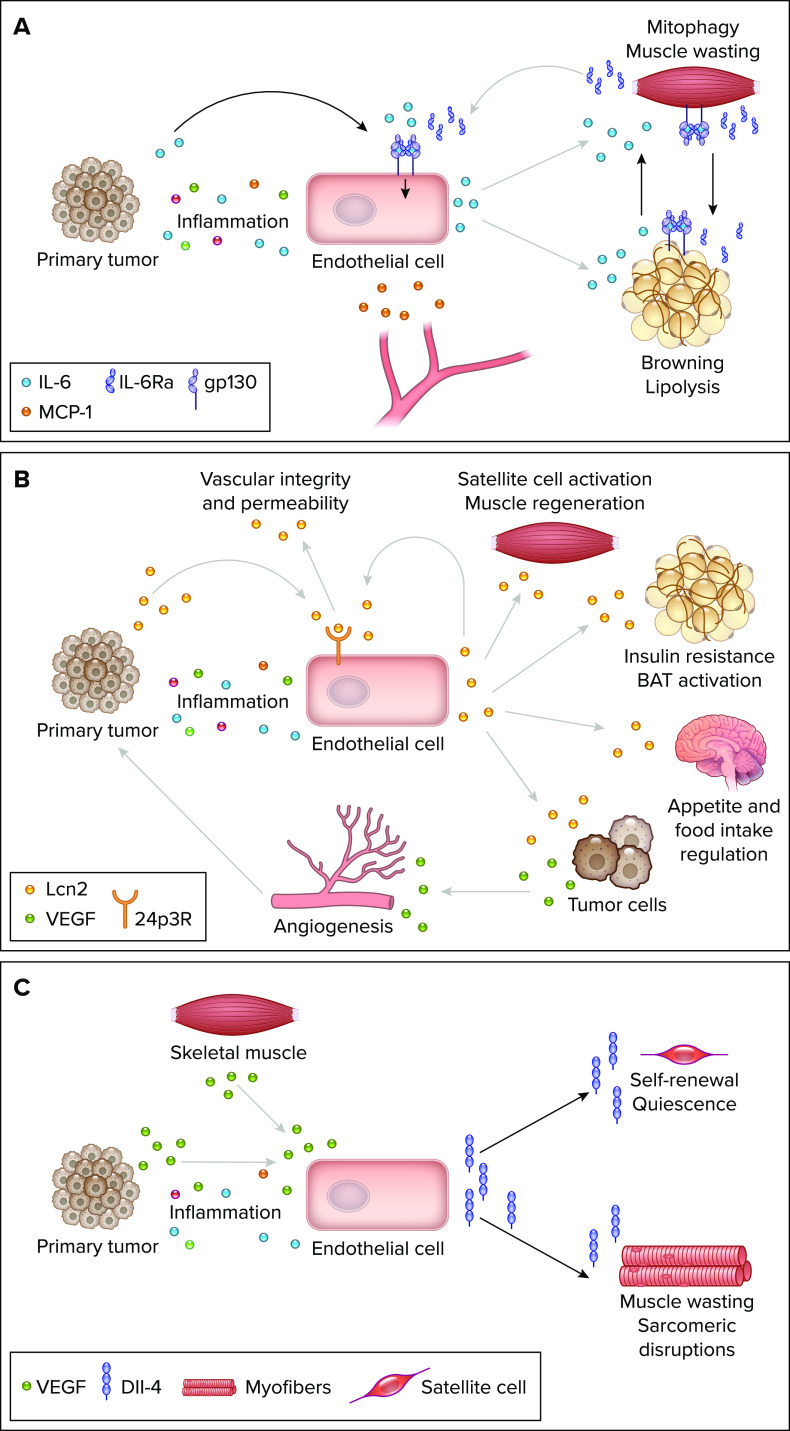
**Endothelial cell signaling pathways during cancer-associated cachexia** *A*: tumor inflammation as well as tumor-derived IL-6 induce endothelial cell (EC) activation and the expression of IL-6 and monocyte chemoattractant protein-1 (MCP-1). Endothelial-derived IL-6 may trigger trans-signaling in adipose tissue causing browning and lipolysis as well as IL-6 release, which in turn activates classical signaling in muscle leading to muscle wasting via mitophagy. Activated skeletal muscle secretes soluble IL-6Rα, which is necessary for trans-signaling in endothelial cells and adipocytes. *B*: tumor-derived lipocalin-2 (Lcn2) and inflammatory factors may induce Lcn2 signaling in ECs via the 24p3R receptor increasing vascular integrity and decreasing permeability. However, EC activation can also induce Lcn2 expression in ECs, which may cause satellite cell activation and muscle regeneration, insulin resistance, and brown adipose tissue (BAT) activation as well as reduced appetite and food intake. Additionally, Lcn2 induces VEGF release from tumor cells which promotes (tumor) angiogenesis. *C*: VEGF from cancer cells and myofibers as well as other inflammatory stimuli induces Dll-4 expression and release from ECs. Dll-4-Notch signaling in satellite cells supports their self-renewal and quiescence. In myotubes, Dll-4 signaling causes muscle wasting and disruptions of the sarcomere structure. Image was created with BioRender.com, with permission.

In 2021, Rupert et al. (89) reported that tumor-derived IL-6 induces a cachectic phenotype in adipose tissue and skeletal muscle via classical and trans-signaling. Primary tumor-secreted IL-6 induced soluble IL-6Rα expression in skeletal muscle, which was transported to the subcutaneous adipose tissue. IL-6Rα promoted IL-6 trans-signaling in white adipose tissue causing increased lipolysis, inflammation, and additional IL-6 production. This activated a feed-forward loop by inducing muscular classical IL-6 signaling stimulating the production of IL-6Rα even more. How endothelial trans-signaling may be involved in this process still needs to be investigated. It is plausible that shed IL-6Rα from the muscle not only activates IL-6 signaling in adipose tissue but also stimulates endothelial IL-6 signaling inducing the expression of STAT3 target genes. Furthermore, tumor-derived TNF-α might lead to an additional increase in plasma IL-6 by its release from ECs. The extent of EC-derived IL-6, endothelial IL-6 signaling and its contribution to tissue wasting and cachexia remains elusive (FIGURE 3A). Generally, it is important to take both, the large interactive surface of blood vessels and the capacity of ECs to amplify tumor-derived signals to modify distant tissues into account.

## Endothelial Lipocalin 2 Signaling and Secretion

Lipocalin 2 (Lcn2) is a secreted acute-phase protein that is highly expressed upon inflammatory stimulation during bacterial infection (90), heart failure (91), chronic kidney disease (92), and cancer (93). Depending on the pathological condition, Lcn2 has either been described as an important factor of the immune system being induced by cytokines especially via IL-6-Stat3 signaling (90) or as adipokine, promoting insulin resistance (94). The described effects of Lcn2 expression during cancer progression are controversial and highly depend on the tumor type as well as its tissue expression. The differential functions of Lcn2 as an oncogene as well as tumor-suppressor are beyond the scope of this review and have been discussed in some detail elsewhere (95, 96). Since Lcn2 has been described to be involved in several cachexia-affected organs as well as in cancer, its role during CAC becomes of interest.

Mechanistically, Lcn2 forms a complex with MMP9 increasing its stability and thereby promoting extracellular matrix remodeling. Additionally, Lcn2 is involved in iron transport context-dependently modulating the intracellular concentration of iron (95). Generally, its expression can be induced by insulin as well as many other cytokines like TNF-α, IL-1β, and IL-6. Therefore, Lcn2 is considered to be responsive to metabolic stress, nutrients, and cytokines (97). Lcn2 is involved in brown adipose tissue (BAT) activation and regulates its thermogenic activity. It is highly expressed in brown adipocytes after cold exposure and stimulates the expression of the BAT markers proliferator-activated receptor-γ coactivator-1α (PGC-1α) and UCP1 (98). In muscle, Lcn2 is expressed in satellite cells within the first day postinjury playing an important role in muscle regeneration (99). However, its myogenic expression is enhanced in ob/ob mice suffering from sarcopenia as well as in wild-type mice after high-intensity training (100, 101).

During CAC, Lcn2 has been described to be upregulated in neutrophils and was found in high concentrations in the plasma and the cerebrospinal fluid of cancer patients correlating with increased mortality (102). Additionally, Lcn2 is increased in plasma and heart tissue worsening cardiac damage after cachexia-induced cardiomyopathy (103). Due to its ability to cross the blood-brain barrier (BBB), Lcn2 can also inhibit food intake by activating the paraventricular and ventromedial neurons of the hypothalamus (104). Lcn2 binds to the type 4 melanocortin receptor (MC4R) on the POMC neurons in the hypothalamus inhibiting food intake and appetite. The inhibition of Lcn2 and MC4R improved cancer-induced anorexia as well as CAC symptoms (102, 105, 106). A scRNAseq study by Huisman et al. (107) showed that tumor-derived factors change the microenvironment of the medial basal hypothalamus and increase Lcn2 expression in cachectic mice. Additionally, it led in the pancreatic CAC model to induce EC inflammation and high Lcn2 expression in hypothalamic ECs. Recently, Lemecha and colleagues (108) determined an ameliorated cachectic phenotype after the global deletion of Lcn2 in a PDAC mouse model. Tumor-bearing Lcn2 knockout mice showed reduced adipose tissue inflammation, less muscle atrophy, and decreased thermogenesis in brown adipose tissue. The authors concluded that Lcn2 may act as a mediator between adipose tissue and skeletal muscle during PDAC progression.

Generally, the vascular function of Lcn2 has primarily been studied in the brain. Following TNF-α stimulation, Lcn2 reduced vascular permeability by rescuing the expression of ZO-1 and VE-cadherin in brain ECs without having an effect on neutrophil adhesion (109). The modulation of tight and adherent junctions to maintain the BBB was also observed in a transient middle cerebral artery occlusion model in which Lcn2 as well as its receptor 24p3R were expressed in ECs (110), indicating LCN2 signaling in ECs in this model. Additionally, brain ECs induced Lcn2 expression after LPS challenge, which was diminished in IL-6 knockout mice (111). Treatment with Lcn2 increased migration and capillary-like tube formation in rat brain ECs in vitro supporting the notion that Lcn2 promotes neurovascular recovery (112). In a glioma model, Lcn2 expression was induced by tumor-derived exosomes via JAK-STAT3 signaling in bEnd.3 endothelial cells (113). The increase of Lcn2 leads to increased BBB penetration of nanocapsules delivering therapeutic monoclonal antibodies without disrupting their integrity. Apart from its function in neuroinflammation, de-aminated Lcn2 has been described to accumulate in the aorta and arteries of obese mice inducing endothelial dysfunction and hypertension by impairing EC-dependent relaxation and contractions (114, 115). Furthermore, expression of Lcn2 in human breast cancer caused upregulated VEGF expression via hypoxia-inducible factor-1α (116). Lcn2 potentiated VEGF-induced angiogenesis in vivo and may promote tumor progression by inducing breast cancer angiogenesis. The relation between VEGF and Lcn2 was also described in proliferative diabetic retinopathy (117). Endothelial Lcn2 was upregulated in diabetic retinal injury and Lcn2 silencing attenuated diabetic retinopathy progression (118).

In summary, whether the effects of tumor-induced Lcn2 expression on cancer and cachexia progression are based on direct changes in EC function, angiocrine signaling, and vascular integrity or indirect changes in VEGF expression and inflammation remains to be determined (FIGURE 3B). Lcn2 affects vascular function, but it is also expressed by ECs leading to the question of how Lcn2 acts on ECs. Whether EC-derived Lcn2 acts systemically or tissue- and site-specific remains elusive and opens up the question of how much the cellular origin and location of Lcn2 matter during disease progression. Determining the key players of the interplay between different Lcn2-expressing compartments and ECs would lead to new insights into an important pathway of many pathological conditions, especially CAC.

## Endothelial Notch Signaling During Cancer-Associated Cachexia

Notch signaling is facilitated by the interaction of Notch ligands (Dll-1, Dll-3, Dll-4, Jag-1, and Jag-2) to one of the four Notch receptors (Notch-1–4). The binding of ligand and receptor activates γ-secretase-induced cleavage and the subsequent release of the intracellular NICD domain activating the transcription of Notch target genes. Notch signaling regulates stem cell function during embryogenesis and adult regeneration. Specifically in ECs, Notch regulates angiogenesis defining tip and stalk cell fates by modulating VEGF sensitivity of ECs (119, 120). During tumor growth, Notch signaling promotes tumor angiogenesis, EMT, and self-renewal of cancer stem cells (121, 122). Endothelial Notch signaling increases metastasis and immune infiltration by promoting transendothelial migration (123) as well as increasing the number of cancer stem cells and circulating tumor cells (124). Additionally, Notch signaling in ECs induces an immunosuppressive microevi-ronment by educating tumor-associated macrophages via CXCL2 (125) making Notch signaling modulation an interesting target for cancer therapy (126).

Notch signaling not only regulates the cancer stem cell pool but also maintains the number of satellite cells (SCs) in the muscle. Notch-3 inhibits SC differentiation and thereby facilitates SC quiescence during homeostasis. However, Notch signaling is also essential during muscle regeneration and the self-renewal of the stem cell pool by regulating the proliferation of SCs after injury (127). Myogenesis is initiated by Notch signaling, which is later replaced by activated Wnt signaling which is necessary for the differentiation in myotubes. The balance of activation and temporal regulation of Notch and Wnt signaling are crucial for effective muscle regeneration (128). The effects of Notch signaling in muscle depend on the cell state leading to controversial results after Notch activation (129, 130). Endothelial Dll-4 is induced by VEGF, which is increased in the plasma in most cancer entities. However, VEGF is also stored in vesicles of skeletal myotubes and can be secreted by exercise, hypoxia, or adenosine release (131). Muscle-derived VEGF regulates the proximity of SCs and ECs, which in turn modulates the quiescent state as well as the self-renewal capacity of SCs via Dll-4-Notch signaling (132). Dysfunctional ECs and impaired angiocrine signaling after metabolic challenge led to less viable and less differentiated human skeletal muscle SCs in vitro (133). Generally, the endothelial-myogenic cross talk, ECs promoting myogenesis and differentiating progenitor cells inducing angiogenesis, is tightly regulated, e.g., by apelin, oncostatin M, and periostin (134).

During CAC, muscle wasting is caused by increased proteolysis as well as defective regeneration mediated by increased Pax7-positive SCs in the skeletal muscle (135). Notch signaling is induced by tumor-derived TNF-α in muscle and tumor tissues during osteosarcoma-induced cachexia. Genetic Notch ablation reduced muscle atrophy and removed the inhibition of muscle differentiation (136). The analysis of sarcoma patient samples revealed decreased muscle regeneration caused by Pax7 and Notch activation in SCs in cachectic versus noncachectic patients (137). Pharmacological inhibition of Notch-Hes1 signaling reduced body weight loss, muscle wasting, and improved survival in a doxo-rubicin-induced cachexia model (138) emphasizing the essential role of Notch signaling in the progression of muscle atrophy. Recently, Fujimaki et al. (139) described that ECs increase Dll-4 release during disuse- and diabetes mellitus-induced muscle atrophy. Upon metabolic and mechanical challenge, the dysfunctional endothelium activated Notch-2 in myofibers leading to reduced muscle mass and disruptions of sarcomere structures. Inhibition of endothelial Dll-4, as well as Notch-2 inactivation in myofibers, prevented muscle wasting induced by metabolic stress (139).

How tumor-derived factors change the EC-muscle cross talk and how exactly Dll-4 expression is induced in ECs during CAC remain to be studied (FIGURE 3C). The maintenance of EC health and the homeostasis of angiocrine signaling during metabolic challenges like CAC may improve the regeneration of muscular function and thereby increase patient survival. The precise mechanisms and direct impact of the endothelium on muscle wasting during cachectic challenges have yet to be elucidated, but the above-mentioned studies implicate a close relation between EC health and muscle regeneration.

## Conclusions and Perspectives

The notion that cancer is a systemic disease highlights the importance of designing therapies that target systemic signaling mechanisms to ameliorate systemic cancer-associated changes. ECs affect the local microenvironment by angiocrine signaling and are key players in many pathological conditions, including cancer. However, ECs can amplify received signals systemically and thereby impact the host’s macroenvironment. Changes in one single vascular bed may translate to other sites or even the whole vasculature (4). Fine-tuning and stabilizing the homeostatic signaling of such a vast reactive surface created by the endothelium could help to revert tumor-induced vascular changes and thereby effectively impact metastasis and cachexia. Determining the correlation between vascular health and patient survival as well as disease progression will create new opportunities in cancer research. The transition from vascular dysfunction to a healthy and quiescent vascular bed has the potential to revolutionize treatment strategies. In addition, there is a need to further study the vascular effects of tumor-derived factors in the future to identify biomarkers of disease progression. This will ultimately guide the stratification of therapeutic regimens to predict and improve the survival of cancer patients.
